# Case report: Cervical spinal cord signal changes in a case of adult-onset subacute sclerosing panenchephalitis

**DOI:** 10.4103/0971-3026.69358

**Published:** 2010-08

**Authors:** Sandeep Sharma, Subhash Kumar

**Affiliations:** Department of Neuroradiology, AIIMS, Ansari Nagar, New Delhi – 110 029, India

**Keywords:** Adult onset SSPE, spinal cord signal changes, brain

## Abstract

In this article, we report a case of subacute sclerosing panencephalitis (SSPE) in which there were central cervical cord signal changes on MRI. The spinal cord is uncommonly involved in SSPE. However, demonstration of spinal cord signal change in a patient of SSPE has significant implications for the differential diagnosis and management.

## Introduction

Subacute sclerosing panencephalitis (SSPE) is a progressive neurologic disorder caused by a defective measles virus.[1] It usually affects children or young adults. The diagnosis of SSPE is based on the characteristic clinical and electroencephalographic (EEG) findings (periodic complexes) and demonstration of elevated antibody titers against measles in the plasma and cerebrospinal fluid (CSF).[2] MRI findings of the brain at different stages of SSPE have been described earlier.[3] The demonstration of spinal cord signal change in a patient with SSPE has significant implications. Although histopathologic and immunologic studies on autopsy cases have demonstrated the involvement of the spinal cord in SSPE,[4,5] the presence of spinal cord signal changes on MRI calls for extensive medical and laboratory workup to rule out other diseases. Spinal cord signal change in a case of SSPE has been described previously in the non-English literature.[6] This case report describes central cervical cord signal changes in a case that was diagnosed as SSPE on the basis of characteristic clinical and EEG findings and the presence of elevated antibody titers against measles in the plasma and CSF. Extensive medical and laboratory workup was done to rule out other diseases.

## Case Report

**Figure 1:**
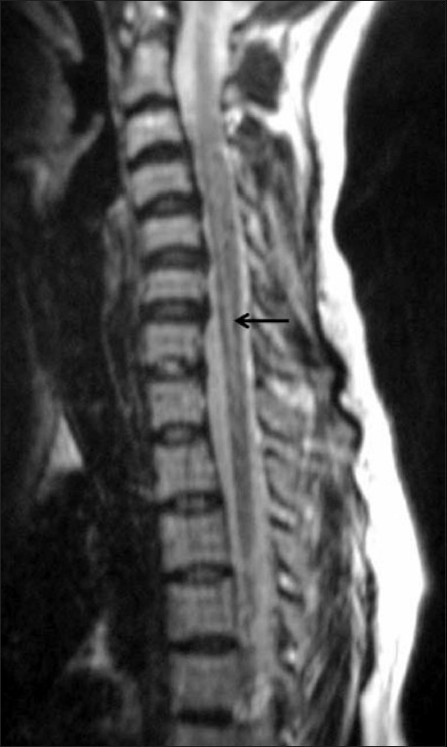
Sagittal T2W MRI image of the cervical spine shows central hyperintensity (arrow) extending from C3 to C7

**Figure 2:**
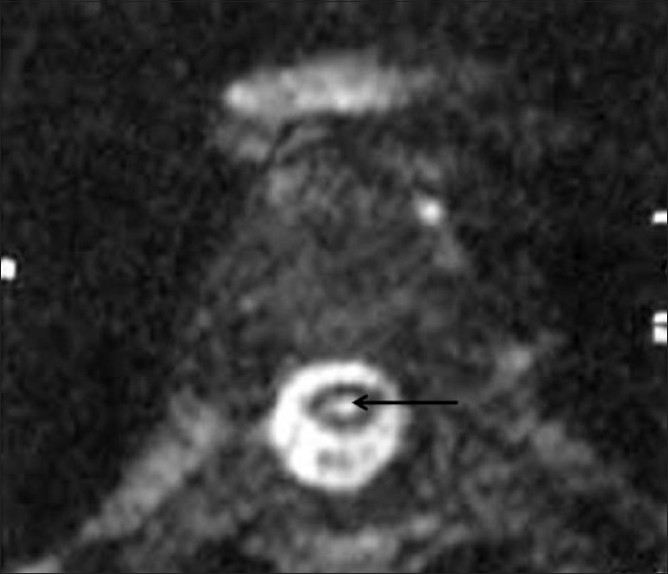
Axial T2W MRI image of the cervical cord better demonstrates the central cervical cord hyperintensity (arrow)

**Figure 3:**
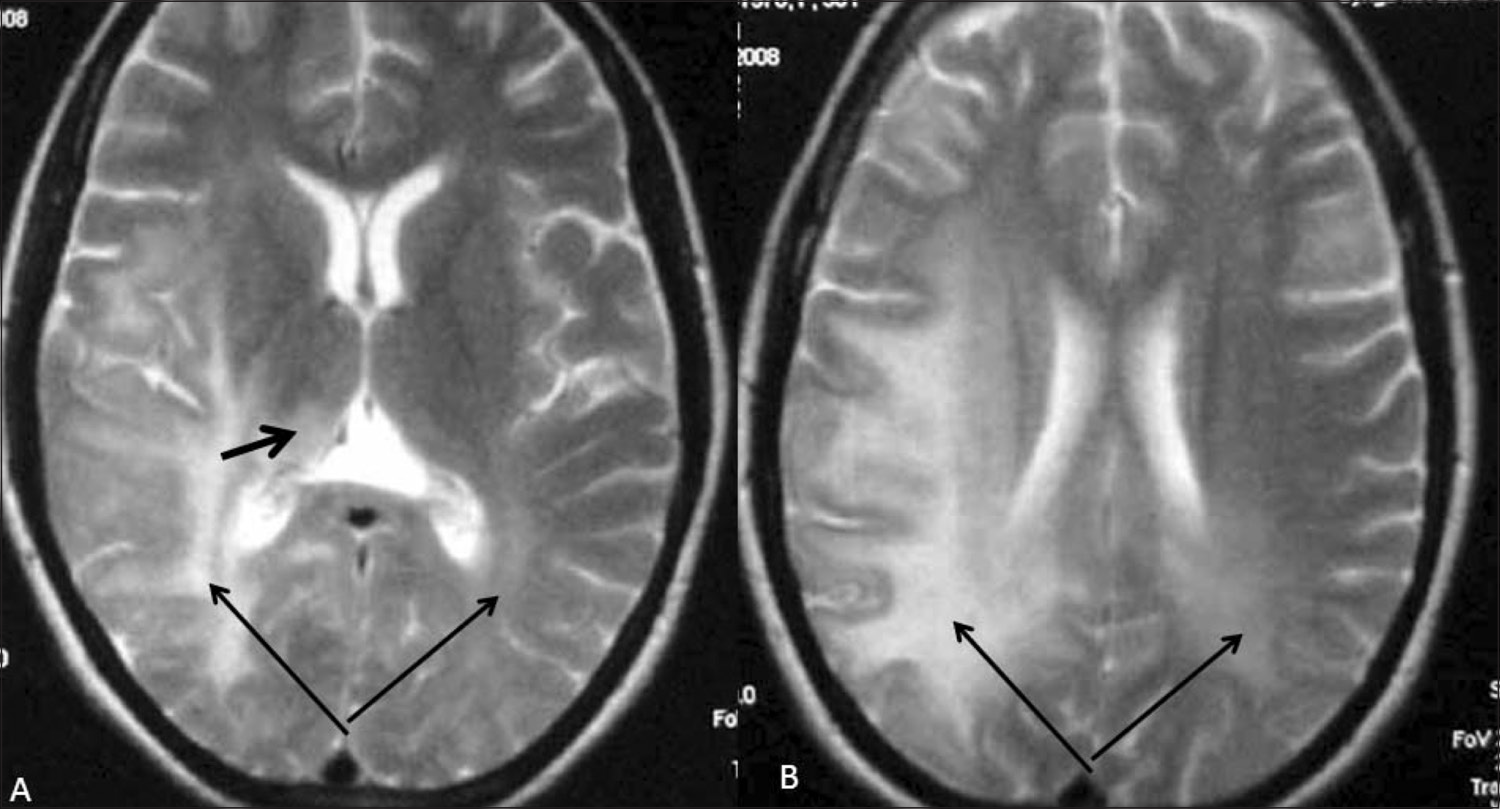
(A and B) Axial T2W MRI images of the brain show bilateral, asymmetric, confluent white matter hyperintensities involving the posterior aspect of the brain, extending from the subcortical white matter (long arrows) to the periventricular white matter. Also noted are the involvement of the thalamus (short arrow in A) and the absence of mass effect or atrophy

**Figure 4:**
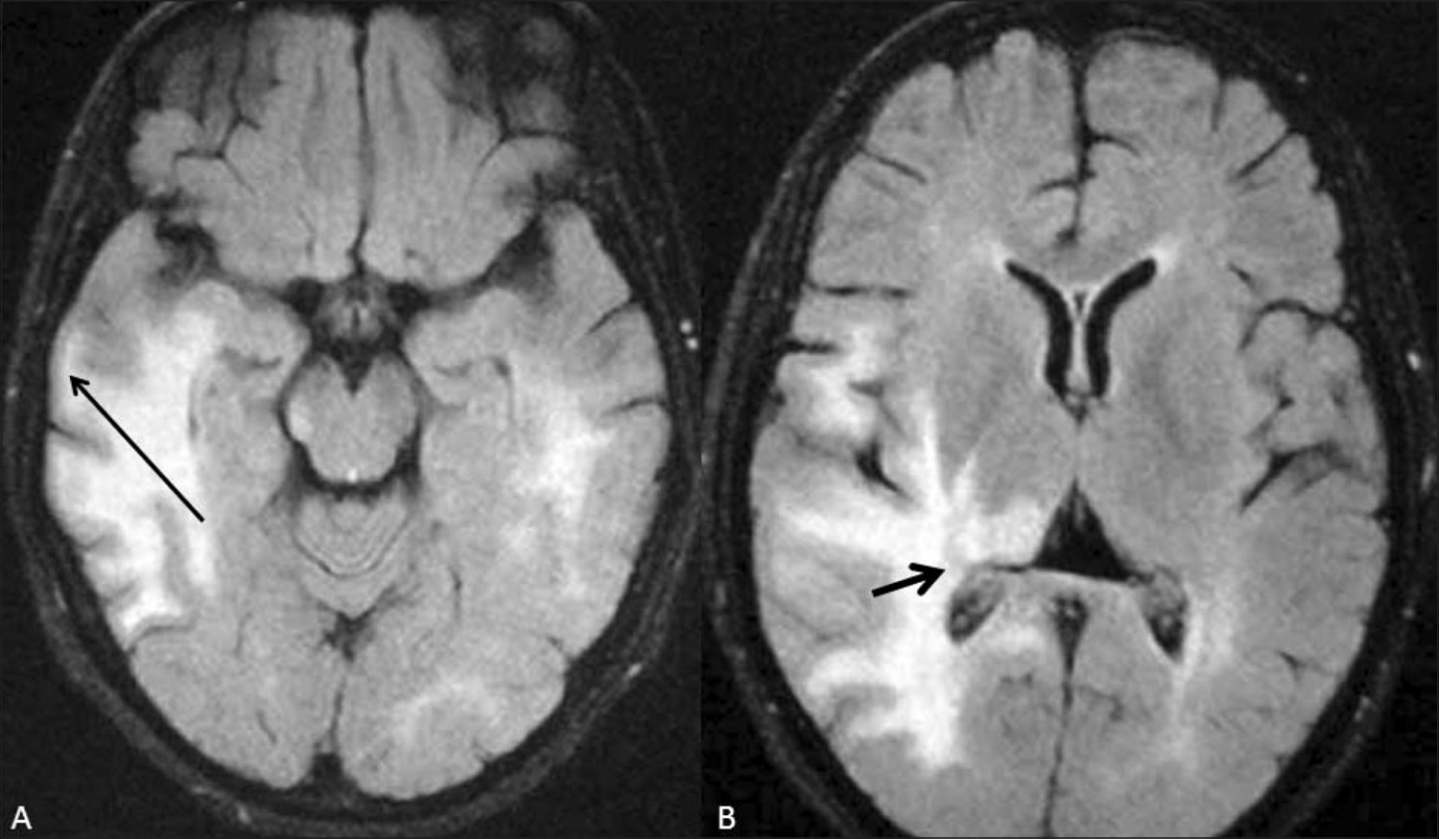
(A and B) Axial FLAIR MRI images of the brain show the T2- hyperintense lesions, seen in Figure 3, more conspicuously, especially in the subcortical (long arrow in A) and periventricular (short arrow in B) regions

A 25-year-old woman presented with a history of sudden visual loss in her right eye 5 years ago, intermittent jerking of the body for 10 months, insidious onset of weakness of the left upper limb 10 months back, weakness of the left lower limb 8 months back, sudden diminution of vision in the left eye 2 months back, and urinary incontinence for 2 weeks. On examination, she was conscious, not oriented to place or time, and was able to identify only her husband. Her speech was interrupted due to myoclonic jerks, which involved the whole body (the left side more than the right), with slow relaxation every 8–10 s. Power was 4/5 on the left side, deep tendon reflexes on both sides were 3+, and the plantars were downgoing. An MRI study of the spine [Figures 1 and 2] and brain [Figures 3 and 4] was performed. MRI of the brain showed large confluent hyperintensities involving the temporal, parietal, and occipital lobe white matter and the right thalamus on T2W [Figure 3] and FLAIR [Figure 4] images. The signal abnormality was asymmetric – being more widespread on the right side – and extended from the subcortical white matter to the periventricular white matter. No definite evidence of mass effect or volume loss was evident. MRI of the spine showed a central cervical cord hyperintensity extending from C3 to C7 [Figures 1 and 2]. Although brain MRI was suggestive of the diagnosis of SSPE, the presence of the cervical cord signal led to a battery of investigations for other neurologic diseases that can cause signal changes in both the brain and the cervical spine on MRI. Further investigations included EEG (which revealed periodic discharges every 3–5 s) and serum and CSF anti-measles IgG antibody levels (which gave test values of 2.734 and 1.396 as against control values of 0.524 and 0.258, respectively). Diagnosis of SSPE was made after extensive exclusion of other diseases and on the basis of the clinical presentation, EEG, anti-measles antibody titers, and characteristic MRI brain findings.

## Discussion

SSPE is a slowly progressive inflammatory and degenerative disorder of the brain that is caused by a mutant measles virus.[1] Although adult-onset cases have been sporadically reported in the literature,[7] the disease typically affects children and adolescents, with the usual age of onset being 5–12 years.

Although MRI neither is essential for diagnosis nor shows correlation with the clinical progression, the involvement of the brain on MRI and its progression are fairly characteristic. Based on the degree of white matter changes and atrophy, Brismar *et al*. classified radiological changes into six stages. Their discussion on neuropathologic findings in SSPE suggests that the disease initially affects the occipital cortex and then progresses to involve the frontal cortex until, finally, it involves the subcortical white matter, brain stem, and spinal cord.[3] There have been a few case reports showing signal abnormalities in the basal ganglia[8] and the pons.[9,10]

A review of literature revealed only one case of fulminant SSPE in whom cervical cord signal changes were present.[6] A 20-year follow-up of another SSPE patient revealed cervical cord atrophy without cord signal changes on MRI.[4]

Allen *et al*. studied the distribution of the measles virus antigen and genomic RNA in the CNS. They suggested that viral spread is usually in a cephalocaudal direction. In two of their short-duration patients, viral RNA was found involving the large neurons in the cervical cord, including the anterior horn cells. In long-duration patients, they found an abundance of viral RNA in all regions of the CNS.[5] In the case presented here, MRI of the cervical cord revealed hyperintensity involving the central aspect of the cord, corresponding to the gray matter.

Adult-onset SSPE has several atypical features,[7] and MRI of the spinal cord is generally performed to rule out other conditions considered in the differential diagnosis. The presence of spinal cord signal change in such cases may delay the diagnosis of SSPE.
